# Developmental competence of Dromedary camel oocytes fertilized *in vitro* by frozen-thawed ejaculated and epididymal spermatozoa

**Published:** 2016

**Authors:** T. H. Scholkamy, D. A. El-Badry, K. Gh. M. Mahmoud

**Affiliations:** 1 Department of Field Investigations, Animal Reproduction Research Institute, Agriculture Research Center, Giza, Egypt;; 2 Department of Artificial Insemination and Embryo Transfer, Animal Reproduction Research Institute, Agriculture Research Center, Giza, Egypt;; 3 Department of Animal Reproduction and Artificial Insemination, National Research Center, Dokki, Giza, Egypt

**Keywords:** Camel, Ejaculated semen, Epididymal spermatozoa, *In vitro* fertilization

## Abstract

The present study aimed to compare the *in vitro* fertilizing capacity of frozen-thawed ejaculated and epididymal spermatozoa in order to standardize the semen preparation protocol for camel *in vitro* fertilization (IVF). Semen samples were collected from 7 Dromedary camels by means of artificial vagina (AV). Ten cauda epididymes were obtained from slaughtered adult camels, isolated, incised and rinsed for obtaining the sperm rich fluid. Ejaculated and epididymal spermatozoa were processed for cryopreservation. Fresh and frozen-thawed ejaculated and epididymal spermatozoa were evaluated for motility, livability, membrane and acrosomal integrities. Frozen-thawed ejaculated and epididymal spermatozoa were used to fertilize camel mature oocytes *in vitro*. The results showed that, the progressive motility of freshly collected epididymal spermatozoa was significantly (P<0.05) higher than ejaculated spermatozoa (49.25 ± 1.75 vs. 38.50 ± 1.50%, respectively). The viability index of epididymal spermatozoa was significantly (P<0.05) higher than that of ejaculated spermatozoa (96.63 ± 2.45 vs. 84.00 ± 4.08, respectively). The post-thaw acrosome and membrane integrities of epididymal spermatozoa were significantly (P<0.05) higher than those of ejaculated spermatozoa. Morula and blastocyst rates of camel oocytes *in vitro* fertilized by frozen-thawed epididymal spermatozoa (59.4 ± 0.8, 19.12 ± 0.7 and 10.29 ± 0.7%, respectively) were significantly (P<0.05) higher than those fertilized by frozen-thawed ejaculated spermatozoa (48.27 ± 3.1, 11.63 ± 1.1 and 5.43 ± 0.8%, respectively). In conclusion, the Dromedary camel frozen epididymal spermatozoa have the capacity to endure cryopreservation, fertilize oocytes and produce embryos *in vitro* better than ejaculated sperm.

## Introduction

 The camel is an important livestock species uniquely adapted to hot and arid environments. The interest in developing assisted reproductive technologies and cryopreservation for the conservation of camel genetic resources has recently increased. The epididymal sperm from slaughtered or recently died animals will increase the opportunities to create semen and to establish their use for artificial insemination (AI), *in vitro* fertilization (IVF), or intracytoplasmic insemination (Turri et al., 2013 ▶; El-Sayed et al., 2015 ▶).

 There are only scarce reports about the use of stored ejaculated semen for IVF in dromedaries because of the difficulties in semen collection, the gelatinous nature of ejaculated semen and the lack of suitable extenders for its storage. Keeping these problems in view, the use of epididymal spermatozoa could be an alternative. However, availability of viable and functional spermato-zoa during the storage period is a prerequisite for AI and IVF, thus necessitating the need for proper storage conditions to maintain the quality and fertilizing ability of the camel spermatozoa for longer periods (El-Badry et al., 2015 ▶).

 Collection and freezing of epididymal sperm samples has been successfully performed in different species of domestic animals: bulls (Chaveiro et al., 2015 ▶), buffalo (Lambrechts et al., 1999 ▶), ram (Kaabi et al., 2003 ▶), bucks (Turri et al., 2014 ▶), equine (Barker and Gandier, 1957 ▶), dogs (Yu and Leibo, 2002 ▶), cats (Axner et al., 2004 ▶), and camels (El-Badry *et al*., 2015) with the aim of developing techniques suitable for storage of genetic material from these animals.

 Freshly collected (Wani et al., 2005 ▶), cooled-stored (Badr and Abdel-Malak, 2010 ▶) and frozen-thawed (Abdoon et al., 2013 ▶; El-Badry et al., 2015 ▶) camel epididymal sperm as well as fresh (Khatir et al., 2007 ▶) and frozen-thawed (El-Sayed et al., 2012 ▶) ejaculated spermatozoa have been successfully used for *in vitro* production of camel embryos. To the best of our knowledge, there was no available report in which the fertilizing capacity of frozen-thawed epididymal as compared to frozen-thawed ejaculated camel spermato-zoa has been investigated. Therefore, the objective of this study was to compare the *in vitro* fertilizing capacity of such spermatozoa in order to standardize the semen preparation protocol for IVF, as an attempt to improve the reproductive efficiency of the Dromedary camel.

## Materials and Methods

**Chemicals**

 Chemicals and media were purchased from Sigma (St. Louis, MO) unless otherwise stated.

**Collection of camel semen**

 Male Maghrabi camels (n=7), 7-2 years of age and 500-650 kg body weight, with a sound history of fertility, raised at Center of Studies and Development of Camel Production, Marsa Matrouh Governorate, Egypt were used in this study. Each camel received 5 kg concentrate feed mixture, 5 kg rice straw and 10 kg green food (*Alfa alfa*) twice daily, while water was offered *ad libitum*.

 Semen was collected during the rutting season (December to April) using bovine artificial vagina (AV; 30 cm long and 5 cm internal diameter; IMV, France). A plastic liner was mounted inside the AV and fixed with two plastic ribbons at both ends. After passing the liner through the AV, 8 cm of cylindrical foam (cut longitudinally) was placed between outer jacket of the AV and liner at the end of the AV. A transparent graded glass water-jacketed Pyrex semen collection tube (IMV, France) was attached to the apex of the internal rubber liner. The AV and the water-jacketed semen vessel were filled with water at temperatures of 40 and 35°C, respectively (Ziapour et al., 2015 ▶). Males were presented to a sexually receptive female, which was physically restrained in sternal recumbency. Once the male mounted the female, the prepuce was directed toward the AV opening.

 Each ejaculate was diluted with equal volume of Shotor (Niasari-Naslaji et al., 2007 ▶) in 50 ml glass bottle with screw cap at 37°C in incubator. Using 5 ml single channel pipette (Eppendorf, Germany) the diluted semen was continuously pipetted for 5 min and the bottles were placed in 100 ml bakers filled with 37°C water and placed for 30 min in 37°C water bath with shaker (Rexmed AI-008, Taiwan) adjusted at 100 RPM. This, in turn, resulted in marked decrease in the viscosity of semen. Just after liquefaction, individual motility and sperm cell concentration were determined by phase contrast microscope and hemocytometer, respectively.

**Collection of epididymal spermatozoa**

 A total of 10 apparently healthy male Dromedary camels, aged between 7 to 10 years, were enrolled in this study during the rutting season. Camel testes were transported from a local abattoir (Kerdasa Abattoir, Giza) to the laboratory in normal saline solution (NSS). Testes were washed with sterile NSS. The cauda epididymides were isolated, incised longitudinally and rinsed 3-4 times with 2 ml of Brackett and Oliphant (BO) medium in 60 mm petri dishes (Bacto Laboratories, Liverpool, Australia) placed on heated stages (37°C).

**Cryopreservation and thawing of ejaculated and epididymal sperm**

 Fluid rich in spermatozoa collected from cauda epididymides and ejaculated semen were diluted with Shotor (Tris-based egg yolk extender, Niasari-Naslaji et al., 2007 ▶) at 37°C in incubator in an appropriate dilution rate to obtain a final concentration of 40 × 10^6^ sperm cell/ml. Diluted samples were then cooled slowly to 5°C in a cooling cabinet for a period of 1.5 h, loaded in 0.25 ml straws (IMV, France) and placed 4 cm above liquid nitrogen in the vapor phase in a foam box for 15 min before being plunged into the liquid phase (El-Badry et al., 2015 ▶). Straws were stored in liquid nitrogen until thawing at 37°C in a water bath for 30 s.

**Evaluation of the ejaculated and epididymal spermatozoa**

 Freshly harvested and frozen-thawed spermatozoa were evaluated for total and progressive motilities under phase-contrast microscope. Sperm viability, abnor-malities and acrosomal status were evaluated by a dual staining procedure (Didion et al., 1989 ▶). For membrane integrity, the procedure described by Jeyendran et al. (1984) ▶ was used to determine the percentage of hypo-osmotic swelling (HOS) positive sperm cells in each semen sample.

**Collection and maturation of oocytes**

**Fig. 1:**
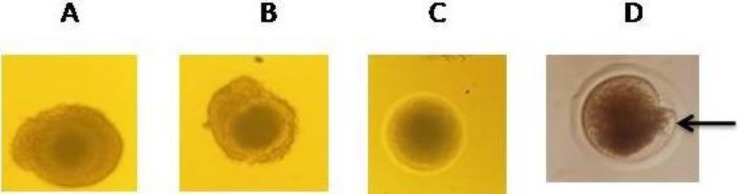
Immature camel oocytes including grade (A), grade (B) and grade (C) and matured camel oocytes with first polar body extrusion (D

 Camel ovaries were transported from a local abattoir to the laboratory in a thermo container containing PBS at 30°C. Cumulus oocyte complexes (COCs) were aspirated from follicles 2-mm in diameter using 18-gauge needle attached to 10 ml syringe. After being washed 3 times in PBS, COCs with at least 2-compact layers of cumulus cells and a homogeneous cytoplasm were selected (grade A and B; Figs. 1A and B) and washed 3 times in maturation medium. For maturation, COCs were cultured in 100 µL droplets of maturation medium (10-15 oocytes per droplet) covered with mineral oil for 30 h (Khatir and Anouassi, 2006 ▶) at 38.5°C in 5% CO_2_ and humidified air. The maturation medium consisted of TCM-199 supplemented with 0.1 mg/ml L-glutamine, 0.8 mg/ml sodium bicarbonate, 0.25 mg/ml pyruvate, 50 µg/ml gentamicin, 10 µg/ml bFSH, 10 µg/ml bLH and 1 µg/ml estradiol in addition to 20 ng/ml of epidermal growth factor (Wani and Wernery, 2010 ▶).

***In vitro*** **fertilization**

 Frozen-thawed spermatozoa collected by AV and from cauda epididymides were prepared for IVF as described by Niwa and Ohgoda (1988) ▶. Briefly, the spermatozoa were washed by centrifugation (800 g for 10 min) in BO medium without bovine serum albumin (BSA) and containing 10 mg/ml heparin and 2.5 mM caffeine (Brackett and Oliphant, 1975 ▶). The sperm pellets were diluted with BO medium containing 20 mg/ml BSA to adjust the concentration of spermatozoa to 2.5 × 10^6^ sperm/ml. Matured oocytes were washed 3 times in BO medium containing 10 mg/ml BSA and were introduced into 100 µL droplets of sperm suspension (about 10-15 oocytes/droplet) under paraffin oil, the spermatozoa and oocytes were co-cultured for 5 h under the same culture conditions (5% CO_2_, 38.5°C, 95% humidity). After that the oocytes were washed in TCM-199 to remove attached spermatozoa. Groups of 10-20 oocytes were again replaced with previously prepared co-culture 100 µL droplets consisting of TCM-199 + 10% FCS. Oocytes of all groups were *in vitro* cultured for 8 days. The morphological appearance of embryos was evaluated under an inverted microscope (Fig. 1D).

**Statistical analysis**

 Two-way analysis of variance and Duncan’s multiple range tests were done for the obtained data after angular transformation of percentages to their corresponding arcsin values (Snedecor and Cochran, 1989 ▶). Data were analyzed using the 1984-version of Costat (Ecosoft, Inc., USA), and the level of statistical significance was set at P≤0.05.

## Results

 Data regarding the pre-freeze and post-thaw characteristics of spermatozoa collected by AV or from cauda epididymides of Dromedary camels are presented in Table 1. Concerning the pre-freeze and post-thaw sperm livability, no significant differences were found between epididymal and ejaculated camel spermatozoa. The progressive motility of freshly collected epididymal spermatozoa was significantly (P<0.05) higher than that of ejaculated spermatozoa (49.25 ± 1.75 vs. 38.50 ± 1.50%, respectively). The post-thaw total and prog-ressive motility of epididymal spermatozoa at 0, 1, 2 and 3 h post-thaw tended to be higher than those of ejaculated spermatozoa. The viability index of epididymal spermatozoa was significantly (P<0.05) higher than that of ejaculated spermatozoa (96.63 ± 2.45 vs. 84.00 ± 4.08, respectively). The percentage of fresh and frozen-thawed epididymal spermatozoa with intact acrosomes were significantly (P<0.05) higher than those of ejaculated spermatozoa. Cryopreservation of ejaculated camel spermatozoa resulted in about 30.0% reduction in the percentage of normal acrosomes. The percentage of frozen-thawed epididymal with intact membranes was significantly (P<0.05) higher than that of ejaculated spermatozoa (44.55 ± 0.84 vs. 40.40 ± 1.03%, respectively).

 As shown in Table 2, the cleavage rate and the developmental potentials to morula and blastocyst stages of camel oocytes *in vitro* fertilized by frozen-thawed epididymal spermatozoa (59.4 ± 0.8, 19.12 ± 0.7, and 10.29 ± 0.7%, respectively) were significantly (P<0.05) higher than those *in vitro* fertilized by frozen-thawed ejaculated spermatozoa (48.27 ± 3.1, 11.63 ± 1.1, and 5.43 ± 0.8%, respectively).

**Table 1:** Pre-freeze and post-thaw livability, total and progressive motility of spermatozoa collected by AV or from cauda epididymides of Dromedary camels

Parameters		Semen source	Epididymal	Ejaculated
Livability		Fresh	80.85 ± 1.78^a^	78.20 ± 1.07^a^
		Frozen	66.85 ± 1.27^a^	64.40 ± 2.28^a^
Total motility (%)		Fresh	67.40 ± 1.33^a^	64.00 ± 1.87^a^
Progressive motility (%)			49.25 ± 1.75^a^	38.50 ± 1.50^b^
Post-thaw total motility (%)	0 h	Frozen	47.25 ± 1.52^a^	45.00 ± 3.54^a^
	1 h		33.00 ± 1.33^a^	29.00 ± 1.87^a^
	2 h		26.25 ± 1.24^a^	22.50 ± 1.12^a^
	3 h		13.75 ± 1.20^a^	10.00 ± 2.13^a^
Post-thaw progressive motility (%)	0 h		32.50 ± 1.25^a^	29.00 ± 1.87^a^
	1 h		25.50 ± 1.40^a^	21.00 ± 1.87^a^
	2 h		18.00 ± 1.11^a^	15.00 ± 1.50^a^
	3 h		10.50 ± 0.95^a^	7.00 ± 1.23^a^
Viability index			96.63 ± 2.45^a^	84.00 ± 4.08^b^
Normal acrosomes (%)		Fresh	87.40 ± 1.43^a^	79.00 ± 1.23^b^
		Frozen	71.25 ± 0.90^a^	56.00 ± 1.92^b^
Swollen spermatozoa (HOS +ve %)		Fresh	58.15 ± 1.28^a^	57.00 ± 1.14^a^
		Frozen	44.55 ± 0.84^a^	40.40 ± 1.03^b^

Means with different alphabetical superscripts within row are significantly different at P≤0.05

**Table 2:** Developmental competence of Dromedary camel oocytes fertilized *in vitro* with frozen-thawed ejaculated and epididymal spermatozoa (mean±SE

Frozen semen	Total oocytes inseminated	Cleavage No. (%)	Morula No. (%)^a^	Blastocyst No. (%)^a^
Ejaculated	129	60 (48.27 ± 3.1)^b^	15 (11.63 ± 1.1)^b^	7 (5.43 ± 0.8)^b^
Epididymal	136	80 (59.40 ± 0.8)^a^	26 (19.12 ± 0.7)^a^	14 (10.29 ± 0.7)^a^

Means with different alphabetical superscripts within column are significantly different at P≤0.05

## Discussion

 There was no significant differences between the total motility of freshly collected epididymal and ejaculated spermatozoa in the current study (67.4 vs. 64.0%, respectively), but the differences were significant (P<0.05) in case of progressive motility (49.25 vs. 38.50%, respectively). The viscid nature of the ejaculated camel semen and the fact that camel spermatozoa are entrapped in a gel like substance of the seminal plasma might be the reason for low spermatozoa motility (Wani, 2009 ▶).

 The proportions of totally motile fresh ejaculated spermatozoa (64.00 ± 1.87%) in the present study are higher than 44-61% (Sieme et al., 1990 ▶), 30-50% (Billah and Skidmore, 1992 ▶), 55% (Hasan et al., 1995 ▶) and 56.6 ± 12.7 (Al-Qarawi et al., 2002 ▶), while lower than 71-84% (Wani et al., 2008 ▶) and 78.3 ± 3.97 (Ziapour et al., 2014 ▶) reported in ejaculated semen of Dromedary camel. The proportions of progressively motile spermatozoa (38.50 ± 1.50%) in the present study are lower than 51.2 ± 10.2 (Al-Qarawi et al., 2002 ▶) and 44.3 ± 6.41 (Ziapour et al., 2014 ▶) reported in ejaculated semen of the same species. These differences could be attributed to individual variation, different extenders, method of collection and liquefaction of semen, season, nutritional status, … etc.

 The post-thaw motility of epididymal spermatozoa was slightly higher (but not significant) and their viability indices were significantly (P<0.05) higher than those of ejaculated spermatozoa. Similar findings of higher post-thaw motility of epididymal spermatozoa were recorded in ram (Garcia-Alvarez et al., 2009 ▶), stallion (Monteiro et al., 2011 ▶) and boar (Rath and Niemann, 1997 ▶). Because ejaculated and epididymal samples were not collected from the same camels, it is possible that the different populations in our study might have differed in sperm quality.

 In the present study, the post-thaw acrosome and membrane integrities of epididymal spermatozoa were significantly (P<0.05) higher than those of ejaculated spermatozoa. Cryopreservation processes are known as being damaging to the sperm cells, and to compromise the integrity of acrosomal structures (Wakayama and Yanagimachi, 1998 ▶). Reyes-Moreno et al. (2002) ▶ claimed that bovine epididymal epithelium fluid from cauda epididymides was able to protect sperm against oxidative damage. Similar to our findings, Varisli et al. (2009) ▶ concluded that acrosomal integrity of ram epididymal sperm was unaffected by chilling stress but the acrosomal integrity of ejaculated sperm was reduced 40% to 50%. Contrarily, Heise et al. (2011) ▶ reported that, there was no difference in the percentage of viable acrosome intact sperm noted between epididymal sperm (60%) or ejaculated sperm (64%) of the same stallions following cryopreservation.

 As compared with previous studies in Dromedary camel, using frozen-thawed epididymal spermatozoa, the cleavage rate recorded herein (59.4%) was more or less similar to that reported by Wani (2009) ▶ (43-60%), and higher than other results (15-32%, Nowshari and Wani, 2005; 37.68%, Badr and Abdel-Malak, 2010 ▶; 26.8%, Moawad et al., 2011 ▶; 25.37%, Fathi et al., 2014 ▶) using freshly collected epididymal spermatozoa. In the present study, the cleavage rate of camel oocytes fertilized by frozen-thawed ejaculated spermatozoa (48.27%) was markedly higher than the cleavage rates of 17-20% reported by Abdoon et al. (2007) ▶, El-Sayed et al. (2012 ▶, 2015) using frozen-thawed ejaculated camel spermato-zoa, and lower than the rates of 64-68% reported by Khatir and Anouassi (2006) ▶ and Khatir et al. (2007 ▶) using fresh ejaculated camel spermatozoa.

 Based on the present data, the cleavage rate of camel oocytes fertilized by epididymal spermatozoa was significantly higher than those fertilized by ejaculated spermatozoa. Similar findings were reported in other animal species like bulls (Katska et al., 1996 ▶; Chaveiro et al., 2015 ▶), goats (Blash et al., 2000 ▶) and boars (Rath and Neiman, 1997 ▶). In contrast, El-Sayed et al. (2015 ▶) in camel found no significant differences between the cleavage rates obtained by epididymal and ejaculated sperm. Also, in cattle, Martins et al. (2007) ▶ recorded lower cleavage rate when using epididymal spermatozoa as compared with the ejaculated ones.

 Using the frozen-thawed epididymal spermatozoa, the morula production rate reported herein (19.12%) was more or less similar to the reported results after IVF of Dromedary oocytes with fresh epididymal spermatozoa (21.32%, Badr and Abdel-Malak, 2010 ▶; 20.0%, Fathi et al., 2014 ▶). Moreover, the use of frozen-thawed ejaculated spermatozoa resulted in 11.63% morula stage which was lower than the rates recorded by Abdoon et al. 2007 ▶ (24.3%) and El-Sayed et al. (2012) ▶ (18.10%).

 In the current study, the blastocyst production rate was 10.29% when frozen-thawed epididymal spermato-zoa were used. Recent reports of using frozen-thawed camel epididymal spermatozoa recorded a blastocyst production rate ranging from 8.3 to 14.8% (Abdoon et al., 2013 ▶; El-Badry et al., 2015 ▶). Fresh epididymal spermatozoa have been previously used for IVF of Dromedary oocytes with a blastocyst production rate ranging from 6 to 24% (Nowshari and Wani, 2005 ▶; Wani, 2009 ▶; Badr and Abdel-Malak, 2010 ▶; El-Sayed et al., 2015 ▶). Using the frozen-thawed ejaculated spermato-zoa, the blastocyst production rate reported herein (5.43%) was within the range reported after IVF of Dromedary oocytes with frozen-thawed ejaculated spermatozoa (4-8%, El-Sayed et al., 2012 ▶, 2015) and was markedly lower than the results after IVF of oocytes with freshly ejaculated camel spermatozoa (23-35%, Khatir and Anouassi, 2006 ▶; Khatir et al., 2007 ▶). The above mentioned variations in the results of *in vitro* developmental potential of camel spermatozoa could be attributed to the method of semen collection, the source of semen, the semen extender, the methods of semen preparation, age of the animals and also the develop-mental competence of the oocytes or media used to culture zygotes.

 In conclusion, it was concluded that the Dromedary camel epididymal spermatozoa have the capacity to endure cryopreservation, fertilize oocytes and produce embryos *in vitro* better than ejaculated sperm
